# Investigation of the Wilson gene ATP7B transcriptional start site and the effect of core promoter alterations

**DOI:** 10.1038/s41598-021-87000-9

**Published:** 2021-04-07

**Authors:** Clemens Höflich, Angela Brieger, Stefan Zeuzem, Guido Plotz

**Affiliations:** 1 Biomedical Research Laboratory, 1st Department of Internal Medicine, University Hospital, Frankfurt, Germany; 2 Medizinische Klinik 1, Biomedizinisches Forschungslabor, Haus 11, Universitätsklinik Frankfurt, Theodor-Stern-Kai 7, 60590 Frankfurt, Germany

**Keywords:** Biochemistry, Genetics, Molecular biology, Gastroenterology, Pathogenesis

## Abstract

Pathogenic genetic variants in the ATP7B gene cause Wilson disease, a recessive disorder of copper metabolism showing a significant variability in clinical phenotype. Promoter mutations have been rarely reported, and controversial data exist on the site of transcription initiation (the core promoter). We quantitatively investigated transcription initiation and found it to be located in immediate proximity of the translational start. The effects human single-nucleotide alterations of conserved bases in the core promoter on transcriptional activity were moderate, explaining why clearly pathogenic mutations within the core promoter have not been reported. Furthermore, the core promoter contains two frequent polymorphisms (rs148013251 and rs2277448) that could contribute to phenotypical variability in Wilson disease patients with incompletely inactivating mutations. However, neither polymorphism significantly modulated ATP7B expression in vitro, nor were copper household parameters in healthy probands affected. In summary, the investigations allowed to determine the biologically relevant site of ATP7B transcription initiation and demonstrated that genetic variations in this site, although being the focus of transcriptional activity, do not contribute significantly to Wilson disease pathogenesis.

## Introduction

The ATP7B gene encodes a copper transmembrane transporter vital for copper homeostasis. It is primarily expressed in liver and brain. In cells of these tissues, Cu^+^ ions are imported into the cytoplasm by the CTR1 protein. From there, ATP7B facilitates its transport in two directions: (1) into the trans-Golgi network for loading copper into proteins that require it, like the serum copper redox protein ceruloplasmin, which is unstable in the absence of copper; (2) from the liver into the bile canaliculus for the purpose of excretion^1^.

Wilson disease is an autosomal recessive disorder of copper metabolism caused by inactivating mutations in the ATP7B gene (Omim # 277900). Deficiency in ATP7B causes accumulation of copper in liver and brain due to hampered excretion of excess copper, causing hepatic or neurological clinical symptoms. The global prevalence has originally been estimated to be 1 case in 30.000 individuals^2^, while higher fractions of 1:7000 with 2.5% individuals carrying a causative allele have been reported in more recent analyses^1,3^. Untreated, the syndrome is fatal, but responds well to copper-reducing therapy if diagnosis occurs in time.

Timely diagnosis is frequently hampered by the phenotypical variability of Wilson disease. Patients present with either hepatic pathology or neurological dysfunction. The age of disease onset is mostly in the 2nd or 3rd decade of life but can vary broadly and may occur in infancy as well as in old age^4^. Phenotypic variation does not correlate well with genotype; inactivating mutations like nonsense and frameshift mutations confer a more severe phenotype than the missense alteration His1069Gln in terms of age at disease onset and ceruloplasmin plasma concentration^5–8^. This is congruent with the observation that His1069Gln does not confer a complete loss of transport activity^9^. His1069Gln and Met769Val, another partially active ATP7B variant, are common in European Wilson patients, making up almost half of all detected variants^10^.

**Figure 1:**
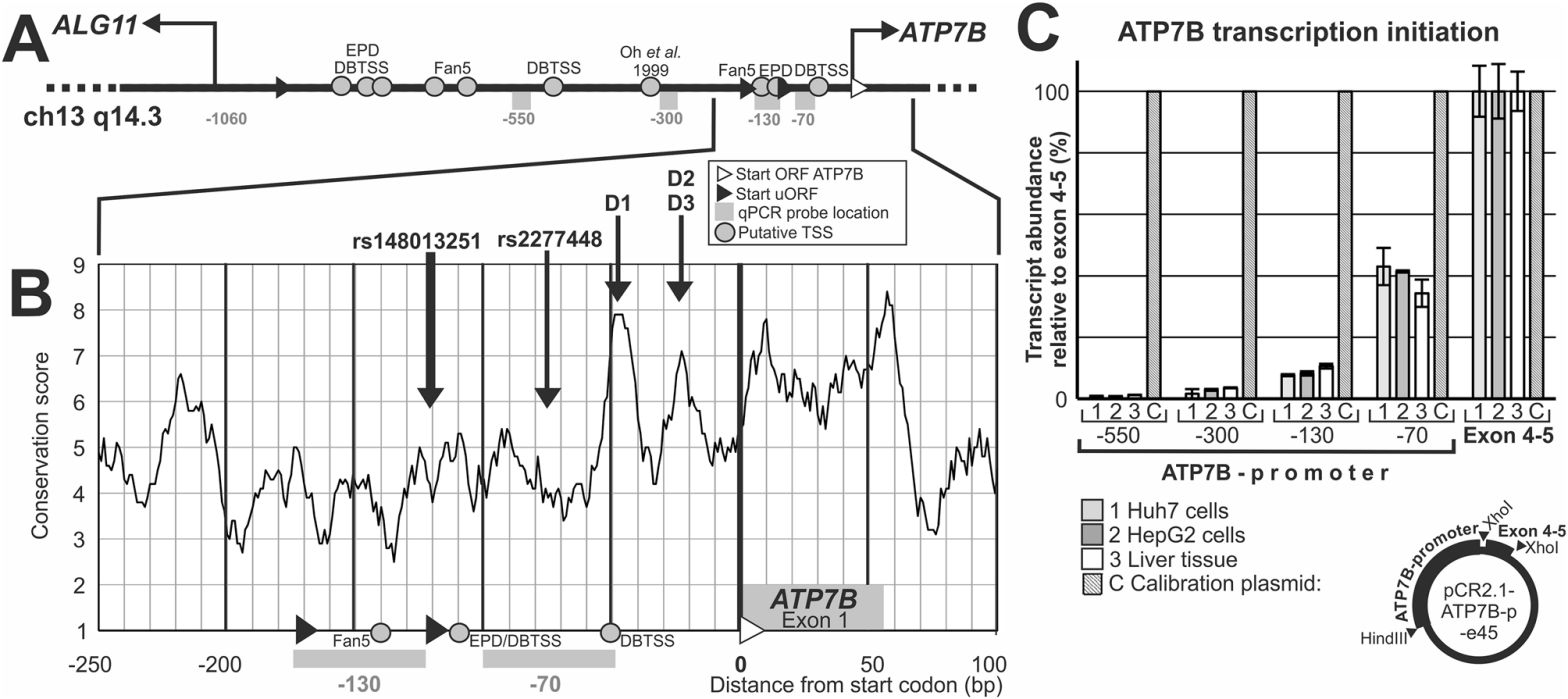
Promoter of ATP7B and transcription initiation. (**A**) Scheme of the bidirectional promoter of the ALG11 and ATP7B genes, which contains several putative transcriptional start sites (grey circles). It also contains three uORFs (black triangles) in direction of ATP7B which are not in frame with the ATP7B translational start site (white triangle). The position of four qPCR probes (− 70; − 130; − 300; − 550) is shown by grey boxes. (**B**) Conservation was scored within the immediate surrounding of the ATP7B translational start site (− 250 to + 100) using ConSeq with appropriate alignments. Two common polymorphisms are indicated, as well the positions of uORFs, putative transcriptional start sites, qPCR probe positions (same symbols as in A.) (**C**) Transcription initiation was tested by quantifying transcript with qPCR probes testing four ATP7B promoter locations (− 70, − 130, − 300 and − 550 bp) in comparison to the coding sequence (Exon 4–5). A calibration plasmid containing both the promoter and Exon 4–5 sequences was used for signal standardization. cDNA from liver cell lines (Huh7, HepG2) and from human liver tissue were tested. DBTSS: Database of Transcriptional Start Sites; EPD: Eukaryotic Promoter Database; Fan5: FANTOM5; uORF: upstream open reading frame.

The majority of causative Wilson disease mutations has been identified in the protein coding sequence of ATP7B or its splicing sites, while data on a role of promoter alterations on Wilson disease are comparatively rare. The 1.3 kbp promotor of ATP7B has been cloned in 1999^11^. It is a bidirectional promoter located between the ATP7B and ALG11 genes (Fig. 1A). A 15 bp deletion at − 441, which is a founder mutation in Sardinia, has been linked to Wilson disease^12^, and two more proximal alterations in the promoter (− 215A > T and − 133A > C) have been described to reduce promoter activity in reporter assays^13^. Some potential transcription factor binding sites for metal-responsive transcription have been identified and been shown to affect ATP7B transcription^14–16^.

Despite these investigations, data on the core promoter of ATP7B, as defined by the transcription start site (TSS), are heterogeneous (Fig. 1A): some evidence suggests two major clusters of transcription initiation, one rather close to the translational start (< − 200) and a distant one (− 600 to − 800)^17^. Both are also supported by experimental data listed in the Database of Transcriptional Start Sites (DBTSS). Further evidence exists for a TSS at − 500 (DBTSS) and between − 600 and − 700 (Fantom5). Moreover, a TSS at − 330 has been suggested as well^11^. Transcription initiation in core promoters usually occurs from one focused TSS or several dispersed TSS, or, as seen in most genes, shows a mixture of both patterns^18^. Within the core promotor, the TSS is typically defined by DNA sequence, while the strength of transcription depends on additional factors^19^. For improving the understanding of the ATP7B promoter and its regulation, identification of its core promoter and TSS is of interest.

We therefore aimed at defining the core promoter of ATP7B that is most relevant for transcription initiation. We also characterized human genetic alterations located within or in proximity of this core promoter. These included two frequent polymorphisms whose impact on transcriptional activity was assessed with the idea that they may be able to modify Wilson disease phenotype in carriers of ATP7B mutations that are not fully inactivating.

## Results

### Transcription initiation of ATP7B

ATP7B mRNA transcription is initiated from a 1 kb bidirectional promoter shared with the ALG11 gene (Fig. 1A). Numerous potential binding sites for metal-responsive transcription factors have been annotated based on sequence similarity, and experimental evidence has been assembled for some. Furthermore, two upstream open reading frames (uORFs) are located in the ATP7B promoter and could potentially modify gene expression^20,21^ (Fig. 1B). However, regarding the transcription start site (TSS), one fundamentally relevant information on promoter structure, conflicting data exist: diverse sites have been identified, distributed over the complete promoter region (Table 1 and Fig. 1A). Since transcription initiation occurs not only during formation of regular mRNA, but also for diverse forms of non-coding RNA including eRNA in promoter regions^22^, it is important to verify which TSS represents that of the regular ATP7B mRNA transcript and therefore marks the ATP7B core promoter. For that goal, the most straightforward method is to test how the individual TSSs quantitatively contribute to the regular mRNA transcript.

**Table 1:** Transcriptional start sites reported in the ATP7B promoter.

Position*	Source	Identifier or tissue/cell origin
− 845	Fantom5	
− 800..750	DBTSS	Adult and fetal tissues
− 755	EPD	ATP7B_1
− 678	Fantom5	
− 629	Fantom5	
− 335	Oh et al.^11^	HepG2 liver cells
− 317	DBTSS	Fetal liver
− 140	Fantom5	
− 110	EPD	ATP7B_2
− 110	DBTSS	Adult brain (and cell lines)
− 50	DBTSS	Diverse tissues/cell lines

*Positions are given relative to the translational start codon of ATP7B (NM_000053.4). EPD, Eukaryotic promoter database^23,24^. DBTSS, Database of transcriptional start sites^25^. Fantom5, database of the FANTOM consortium^26^; Oh et al.^11^.

Therefore, we quantified by qPCR the abundance of sequences of ATP7B transcript 5’ to the translational start in different distances (− 550, − 300, − 130 and − 70 bp) as compared to the whole amount of transcript (measured at the junction of exons 4 and 5 of ATP7B) (Fig. 1A,B). We performed this analysis with liver tissue as well as with the common liver model cell lines HepG2 and Huh7 and specifically designed a plasmid for qPCR calibration (Fig. 1C).

Transcription was found to be initiated in immediate proximity of the translational start site, with about 40% of the transcripts being detected with a qPCR probe located at − 70 bp (Fig. 1C). Therefore, transcription initiation from the depth of the ATP7B promoter is negligible, but instead occurs within 130 bp 5’ of the ATP7B translational start. Only two experimental TSS (Table 1) are therefore candidates for transcription initiation of the regular ATP7B mRNA: those documented at − 110 and at − 50. Since transcripts starting at − 50 would not have been detected by the − 70 qPCR probe, additional transcription initiation occurs from the − 50 TSS.

In summary, the ATP7B core promoter is located in immediate proximity of the translational start site (between start site and position − 130 bp) and likely uses two closely spaced TSS for regular ATP7B mRNA transcript formation.

### Alterations in conserved areas of the core promoter can reduce transcriptional activity

Analysis of the core promoter area revealed two spots of elevated average sequence conservation (Fig. 1B). Three human single-nucleotide substitution variants (SNPs) are located in these sequences (Fig. 1B and Supplementary Table 1): − 54G > T (D1; rs115564351) and 36C > T and − 32A > G, corresponding to rs762339422 (D2) and rs759260854 (D3), respectively. These single base pair alterations are rare, therefore clinical information on a potential association with Wilson disease is unavailable. Therefore, their analysis may provide information on their potential contribution to Wilson disease on one hand and allow conclusions concerning the general significance of the conserved sequences on the other hand.

We introduced these alterations into a luciferase reporter plasmid pGL4.10 containing the 1kbp ATP7B promoter sequence. As negative control, the reporter plasmid without promoter was used. Linearity of the reporter assay measurements and homogeneity of the applied plasmid DNA amounts were extensively verified (Supplementary Figure S3).

**Figure 2:**
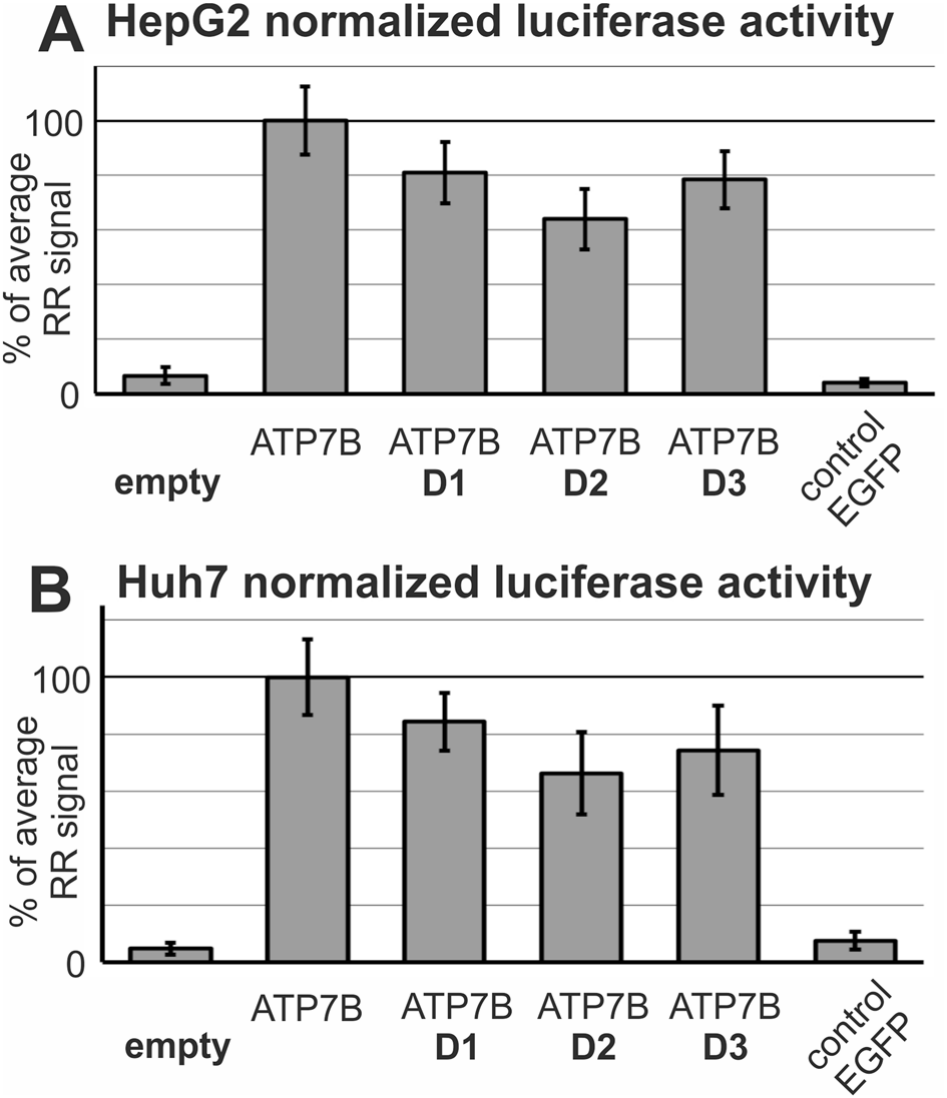
Luciferase reporter activity of genetic promoter variants. The experiment was performed as detailed in Materials and Methods. Expression of the luciferase reporter constructs D1 (rs115564351, − 54G > T), D2 (rs762339422, − 36C > T) and D3 (rs759260854, − 32A > G) in HepG2 or in Huh7 liver cells were tested by co-transfection with *renilla* luciferase standardization vector pGL74. Transfection efficiencies (*renilla* luciferase activity) were comparable in all ATP7B promoter constructs (data not shown). Promoter activity was determined by normalizing *firefly* luciferase activities by renilla luciferase activity for transfection efficiency. Average values of 10–20 independent experiments normalized to reference sequence promoter are shown.

Luciferase measurements were performed in two liver cell lines (HepG2 and Huh7) and demonstrated that all one single base substitutions (D2) conferred a significant (*p* < 0.01) reduction of transcriptional activity, which was 62% of the wildtype sequence (Fig. 2).

### Two frequent polymorphisms are located in the core promoter region

In proximity to the core promoter, two frequent polymorphisms are located (Fig. 1B, Table 2): rs148013251 is a 5-bp-duplication at − 123 bp, and rs2277448 is a single nucleotide substitution at − 75 bp. Overall, both polymorphisms are present in 40% of humans. There is an overrepresentation of the polymorphisms in Europe and Asia and an underrepresentation in Africa (Supplementary Figure S2A).

**Table 2:** Two polymorphisms located in the ATP7B promoter.

Identifier	Description (NG_008806.1)	Description relative to ATP7B start codon	Reported allele frequency	Genotype in 24 healthy individuals		
				Reference (%)	Heterozygous (%)	Homozygous (%)
rs148013251	g.5035_5039dupCGCCG	− 123 duplication CGCCG	0.42 (GnomAD) 0.37 (1000Genomes)	33	38	29
rs2277448	g.5083C > A	− 75 C > A	0.42 (GnomAD) 0.43 (TOPMED) 0.48 (1000Genomes)	17	33	50

To investigate this unequal geographical distribution more closely, we assessed their occurrence in ancient and recent whole human genome sequences of individuals of different geographical origin (Supplementary Table S3). This confirmed that the reference sequence genotype is predominant in present-day humans from Africa. It also suggests that the reference sequence represents the original genotype since it is found in ancient genomes of Neanderthals and Denisovans. However, both polymorphisms seem to have formed early in modern Eurasian humans, since they are already present in the *Ust-Ishim* genome^27^. Since the rs148013251 was present exclusively in individuals carrying also the rs2277448 in all investigated samples (Supplementary Table S3), the rs148013251 may have been acquired as an additional variant in rs2277448 carriers (Supplementary Figure S2B). The present predominant occurrence of these polymorphisms in people of Eurasian ancestry therefore probably originated during or after migration of *Homo sapiens* out of Africa into Eurasia.

### Effect of polymorphisms on promoter activity

**Figure 3:**
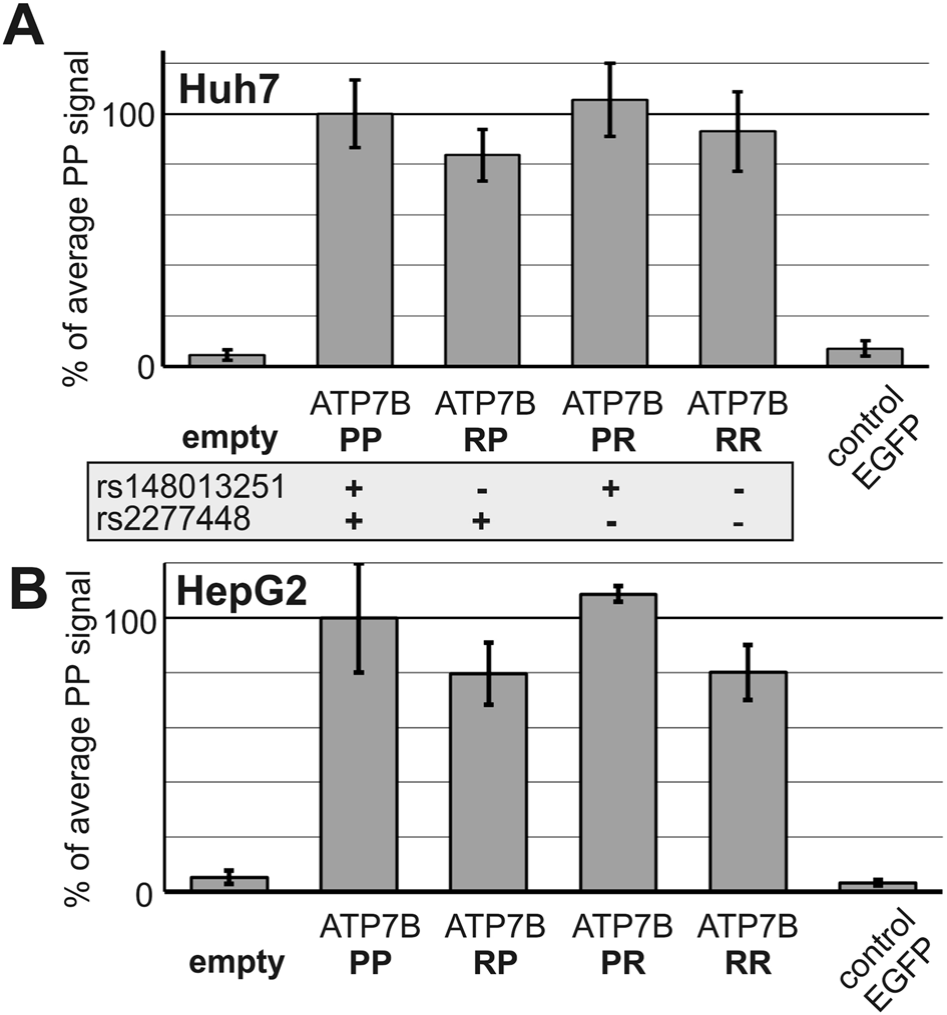
Promoter activity of different polymorphic promoters. The ATP7B promoter was cloned into a pGL4.10 *firefly* luciferase reporter. Expression of the luciferase reporter constructs PP, RP, PR, RR (polymorphic genotypes are indicated in the grey box) was tested in Huh7 and HepG2 liver cells by co-transfection with *renilla* luciferase standardization vector pGL74. Original pGL4.10 vector without ATP7B promoter was also transfected for control (“empty”). After 48 h, signals of both luciferase proteins were scored using the DualGlo system. Transfection efficiencies (*renilla* luciferase activitieswere comparable in all ATP7B promoter constructs (data not shown). *Firefly* luciferase reporter activity was normalized on *renilla* luciferase activity. Average values of 6–12 independent experiments normalized to PP are shown.

The polymorphisms cannot be causative for Wilson disease, but they may still confer changes in ATP7B gene expression. In Wilson disease patients carrying ATP7B mutations with incomplete inactivation of the gene product, the polymorphisms may confer a clinically relevant modification of the phenotype or disease penetrance. The majority of causative Wilson disease alterations confers such incomplete inactivation, these include (predominant location of occurrence is given in brackets): His1069Gln (Europe), Met769Val (UK), Arg969Gln (Greece), Met645Arg (Spain), Gly710Ser (Austria, Turkey) and Arg778Leu (Asia)^9,10,28,29^. We therefore investigated their impact more closely, first by assessing potential effects on transcriptional activity. Luciferase reporter plasmids were constructed containing either the reference promoter sequence (RR), either polymorphisms individually (RP and PR) or both polymorphic sites (PP) (Supplementary Table 1). All four constructs with different polymorphic status showed similarly high expression of the reporter (Fig. 3).

### ATP7B promoter polymorphisms, ceruloplasmin and copper blood levels in healthy individuals

Transcriptional activity is on one hand directly conferred by the (core promoter and enhancer) DNA sequence, on the other hand indirectly by epigenetic effects like histone modifications that alter accessibility of the DNA sequence^19^. While direct effects of DNA sequence alterations on transcription are readily detectable in luciferase reporter assays, epigenetic effects are not reflected in this experimental approach. We therefore tested the biological consequences of the polymorphisms on copper household in 24 healthy probands by determining their polymorphic status and blood copper and ceruloplasmin serum levels.

**Figure 4:**
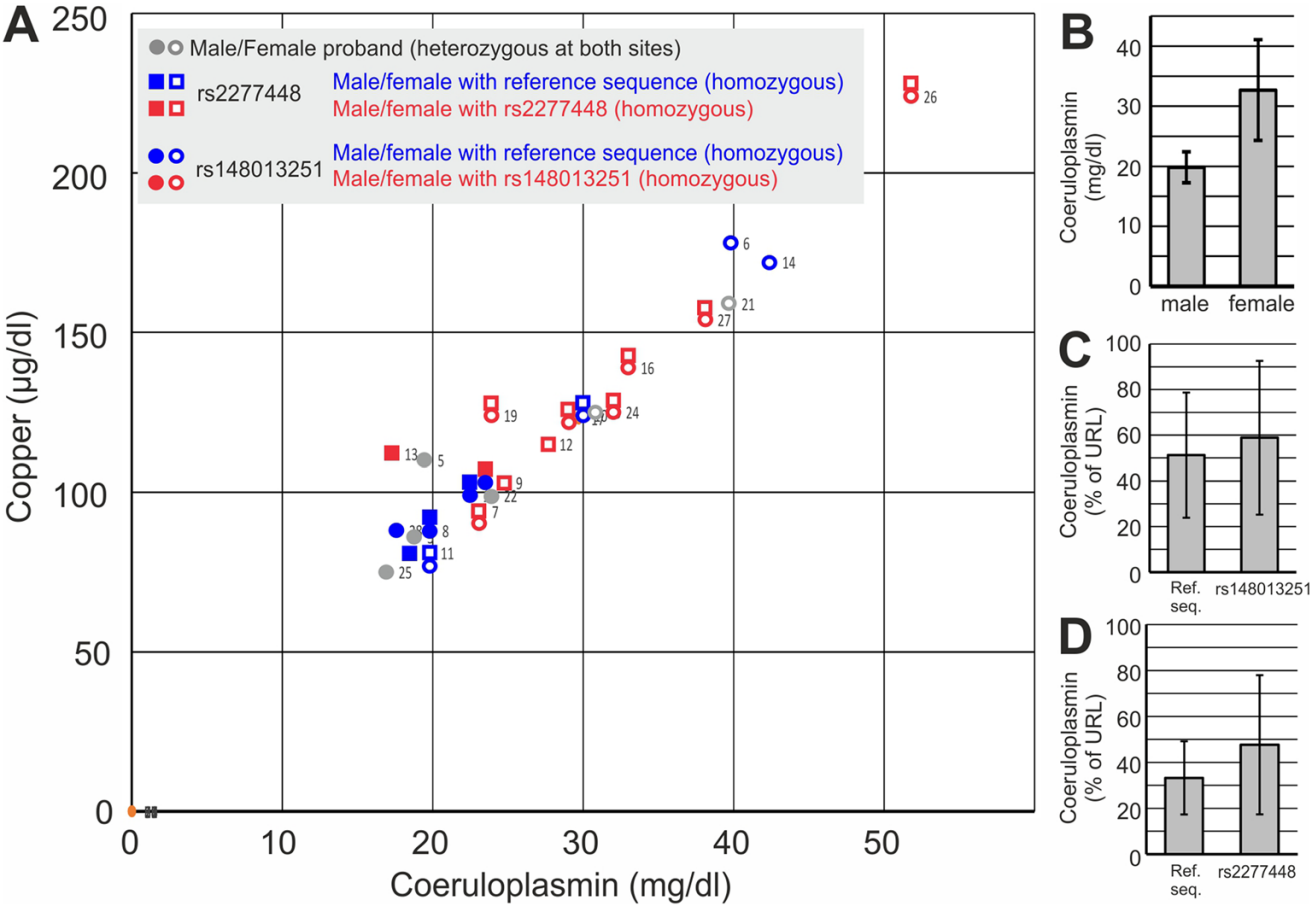
Promoter polymorphisms in relation to coeruloplasmin and copper blood levels. Rs2277448 and rs148013251 genotype, copper and coeruloplasmin serum levels were determined in 24 healthy probands. (**A**) Correlation of genotypes, sex, coeruloplasmin and copper serum levels in all individual probands. (**B**) Sex and coeruloplasmin levels. (**C**) and (**D**) Coeruloplasmin levels as percent value of individual (male and female) upper reference limits in individuals either having homozygous rs148013251 (**C**) or homozygous rs2277448 (**D**) in comparison to respective individuals with homozygous reference sequence (Ref. seq.).

Allele frequencies were 48% and 65% for rs148013251 and rs2277448, respectively, with 50% being homozygous carriers of the rs2277448 (Table 2), consistent with previously reported frequencies. As expected, copper levels correlated well with those of its serum transporter ceruloplasmin (Fig. 4A). No significant association was evident in the comprehensive analysis of both genotypes and copper and ceruloplasmin serum levels. Since ceruloplasmin serum levels are sex-dependent, being significantly higher in women (Fig. 4B), we normalized these differences by adjusting the measured ceruloplasmin levels to their sex-dependent upper reference limit (Fig. 4C,D). However, no significant association of polymorphic status and clinical copper household parameters was detectable.

## Discussion

It was the aim of this work to clarify the location of the ATP7B core promoter (containing its transcriptional start site) and assess the effects of human genetic variants located in this core promoter area. While experimental results from previous investigations and promoter database data have suggested numerous potential TSS for ATP7B dispersed over almost the whole 1 kbp promoter region (Table 1 and Fig. 1A), our approach was to quantitatively determine which of these TSS contribute(s) relevantly to ATP7B transcription.

**Figure 5:**
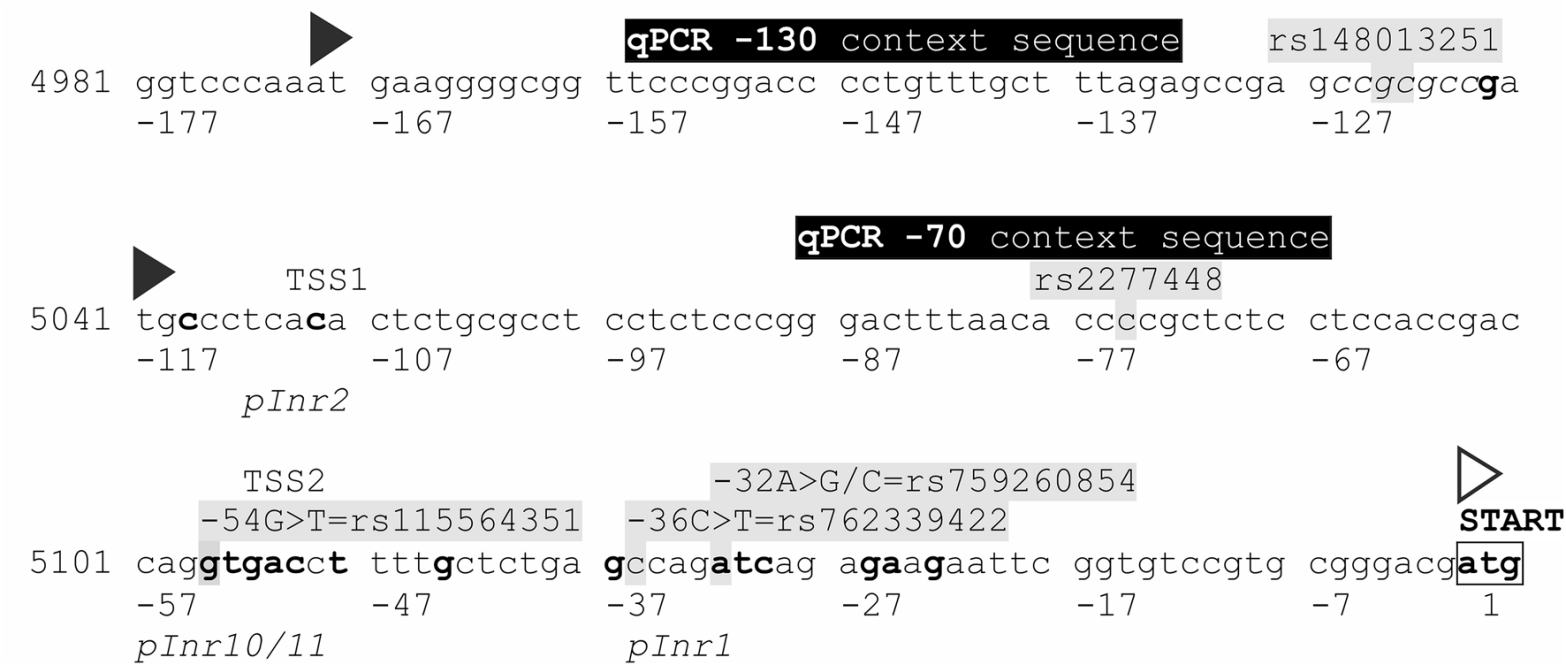
The ATP7B core promoter. Numbers on the left refer to genomic ATP7B reference sequence NG_008806.1, numbers below the DNA sequence are relative positions to the ATP7B start codon. The regular ATG start codon is indicated by an open triangle, irregular start codons (potential uORFs) out of frame by black triangles. Nucleotides in fat and framed are conserved, those with gray shading have been studied as variants in the current analysis. Transcriptional start sites (TSS) as reported in databases are indicated above the sequence. qPCR probe core sequences are indicated in frames above the sequence.

The qPCR assessment showed that the major fraction of transcription initiates in immediate proximity of the translational start site: while only 10% of ATP7B transcript contain sequence at − 130 bp before translational start, it is approximately 40% at − 70 bp, suggesting that the core promoter is located within ≈130 bp before the start codon. Two of the previously suggested TSS are indeed located within this DNA area: one at − 110, one at − 50 (Table 1 and Fig. 5)^23,24,30^. Therefore, other previously identified TSS more upstream do not seem to contribute significantly to transcription initiation of ATP7B^26^.

Some core promoters contain DNA sequences facilitating binding of specific transcription factors like the Initiator-(Inr)-site that binds RNA polymerase II^31^. However, many promoters do not contain such motifs, and some motifs are highly degenerate so that they cannot be reliable recognized^32^. Screening of the ATP7B core promoter for core promoter motifs using the ElemeNT tool^33^ yields, as expected, a multiplicity of potential Inr (pInr) sequences (Supplementary Table 4). The second best, however, is congruent with the first experimental TSS (TSS1:pInr2 in Fig. 5), which could be responsible for the fraction of transcripts detected with the − 70 qPCR probe. Additionally, other potential initiator elements are located in the second TSS (TSS2:pInr10/11), and the best scoring Inr is located further downstream (pInr1). Transcription may either be initiated from these TSS individually or in a “dispersed” mode of transcription which is common in eukaryotic promoters^18^. A prevalent feature of such dispersed promoters are ATG deserts^18,34^. Indeed, ATP7B transcription initiation occurs within a small ATG desert comprising approximately 120 bp (Fig. 5). Consequently, the two ATG start codons (out of frame) at positions − 118 and − 169 are not transcribed and therefore do not confer down-regulation of protein expression which has been observed in many genes^20,21^.

Of three single-nucleotide alterations located in conserved sequences within the ATP7B core promoter, one (D2) caused a significant repression of transcription. D1 (rs115564351; − 54G > T) is rare in most populations but occurs in 2% of Africans^35^, suggesting no relevance for disease. The other two alterations (rs762339422 and rs759260854) are very rare, and clinical carrier information is missing, therefore clinical conclusions are not possible. rs762339422 (D2) displayed the most pronounced effect on expression. D2 has been observed in two Chinese Wilson disease patients without providing clear evidence for clinical relevance^36^ and has been previously suggested to affect gene expression based on prediction^37^. However, since the expression repression was rather moderate, it is unlikely to be causative.

These observations suggest that small alterations in the core promoter do not suffice to abolish transcription enough to cause Wilson disease. This is consistent with the general observation that no causative genetic variants have as yet been reported in the core promoter area.

Two frequent genetic polymorphisms are located in the core promoter area. These represent the majority of ATP7B 5’ UTR entries in the Wilson mutation database since their observation has been reported in many publications^38^. Both are present in approximately half of the human population with a specifically high prevalence in Eurasians; our analysis suggests this may reflect their occurrence during migration of *Homo sapiens* out of Africa. While neither polymorphism can be causative for Wilson disease due to their high prevalence, their location within the ATP7B core promoter potentially enables them to alter ATP7B transcription to some degree, similarly as has been observed for the D2 sequence variant. The majority of Wilson disease patients carry missense alterations that can confer variable, often incomplete effects on protein functionality which consequently show a less severe clinical phenotype in contrast to the more rarely observed truncating mutations^6,8,9,39–42^. Therefore, the two frequent core promoter polymorphisms are candidate genetic modifiers of Wilson disease phenotype. The marked variability of the Wilson disease clinical phenotype that is even observed in carriers of the same mutation(s) has not yet been explained; several modifying influences have been suggested to contribute: besides environmental factors, epigenetic and transgenetic effectors and protein interactions of different missense variants are in the list^40,43,44^.

However, neither luciferase reporter measurements suggested a significant functional impact of the polymorphisms, nor could a modulation of clinical copper household parameters detected. Consequently, a potential modification of Wilson disease phenotype could not be substantiated. However, some restrictions remain: for example, core promoters usually serve the general transcription from a gene, while more distant promoter elements may modify transcription strength^19^, as has been described for ATP7B as well^15^. It has not been determined in this study if the polymorphisms may modulate distant promoter regulation. Moreover, we only investigated copper household clinical parameters as surrogate markers since direct examination of the association of the polymorphisms with ATP7B gene expression would have required a liver biopsy to assay ATP7B mRNA levels of carriers of different polymorphic status, which was not within the scope of ethical approval. The ultimate clinical clarification of the question if these two frequent polymorphisms modify Wilson disease phenotype would require a polymorphism-phenotype analysis in a large cohort of Wilson disease patients with two (ideally identical) causative mutations that have residual activity. However, the current study does not give evidence in favor of a modifying influence.

In summary, we localized the transcriptional start site of the biologically relevant ATP7B core promoter and showed that one human genetic variant located in conserved areas of this core promoter has a reducing effect on ATP7B transcription, presumably without being of clinical effect. Moreover, we clarified the effect of two frequent polymorphisms in the core promoter and confirmed that they are unlikely to contribute to Wilson disease phenotypical variability.

## Methods

### Probands and gDNA analysis

The investigation of the ATP7B genotype and the serum levels of copper and ceruloplasmine in 30 healthy volunteers was approved by the ethics committee of the University Hospital of Frankfurt (Goethe-University), #88/19 in March 2019. All volunteers gave written, informed consent. Peripheral EDTA blood was taken and gDNA was extracted using the DNA mini kit from Qiagen (Hilden, Germany). Copper (photometric assay) and ceruloplasmine (immunological assay) serum levels were determined in the central laboratory of the hospital with a Roche Cobas 8000 analyzer.

### Cell lines

HepG2 hepatocellular carcinoma cells were purchased from DSMZ (Braunschweig, Germany) in December 2019. Huh7 cells were kindly provided by Albrecht Piiper (Universitätsklinik Frankfurt, Germany). Both cell lines were maintained in DMEM with 10% fetal bovine serum and antibiotics.

### Generation of luciferase and qPCR control vectors

pGL4.10 (firefly-luciferase without promoter) and pGL4.74 (renilla luciferase control vector using an HSV-TK promoter) were purchased from Promega, Wisconsin, U.S.A., and the pCR2.1 TOPO cloning vector was from Invitrogen, Carlsbad, U.S.A). The ATP7B gene luciferase promoter was amplified by PCR from Huh7 cell genomic DNA using standard conditions with the primers ATP7B-Promo-XhoI-F (5’-CTCGAGCTgctcacctcaacaacttgca-3’) and ATP7B-Promo-HindIII-R (5’-AAGCTTACggacaccgaattcttctctga-3’), which allowed to generate a fragment comprising 1126 bp of the promoter (NG_008806:4024-5149) with simultaneous introduction of XhoI and HindIII restriction sites appropriate for cloning the fragment into pGL4.10 in frame. The PCR product was subcloned in pCR2.1, resulting in pCR2.1-ATP7B-p. The ATP7B fragment was cut from this plasmid using XhoI and HindIII and transferred to pGL4.10. All constructs were verified by direct sequencing to be correct: the resulting pGL4.10-ATP7B-0 vector contained wildtype ATP7B genetic sequence (NG_008806:4024-5149) except for the presence of the two polymorphisms under investigation (rs148013251, rs2277448) and a single nucleotide variant (rs1427836170) located deep into the ATP7B promoter, 842 5’ of the transcriptional start, within an unconserved region. Further variants of the promoter were subsequently prepared by site-directed mutagenesis using appropriate primers (Supplementary Table 1) with the QuikChange II Site-directed Mutagenesis Kit (Stratagene, La Jolla, U.S.A.).

A calibration plasmid for qPCR measurements was constructed by amplifying an ATP7B gene fragment containing exons 4 and 5 (NM.00053.3:1781-2023) using the primers 5’-agctCTCGAGttctctccgt gttggttgcc-3’ and 5’-agctCTCGAGaataattttgataatatcccgtgg-3’ which introduced two terminal XhoI sites in the product. This product was inserted into the XhoI restriction site of the pCR2.1-ATP7B-p plasmid (see above).

### qPCR

Acquisition of samples, reverse transcription and qPCR have been documented according to the MIQE guidelines^45^. **RNA acquisition and cDNA synthesis.** Total RNA was extracted from growing Huh7 and HepG2 cells using the Trizol reagent (Invitrogen, Germany) according to the manufacturers’ recommendations. Total RNA was dissolved in 30 µl RNAse-free water after ethanol precipitation. Cell harvesting and RNA extraction were performed within two hours, and RNA was subsequently either used directly for cDNA preparation or immediately stored at − 80 °C. In order to reduce handling times, residual genomic DNA was not removed since the reverse transcription was preceded by a DNA digestion (see below). RNA content was quantified on a Beckman DU-800 spectrophotometer. cDNA was created from 2 µg total RNA. This RNA had either been prepared fresh or from samples stored at − 80 °C for less than one month and not thawed more than twice. Since most qPCR probes tested ATP7B in the promoter, they were sensitive for gDNA contamination. Therefore, RNA samples (2.5 µg) were incubated with TurboDNAse I in a total volume of 20 µl at 37 °C for 30 min. Subsequently, DNAse I enzyme was removed with 2 µl DNAse inactivation reagent according to the manufacturers’ instructions (both reagents included in the Turbo DNA-free kit, Invitrogen, Carlsbad, U.S.A.). For assessment of the transcript levels in liver, we used a human liver RNA preparation (FirstChoice, #AM7960 LOT 2021608, ThermoFisher, Massachusetts, U.S.A). All DNA-free RNA preparations (2 µg) were incubated with 1 µg random hexamer primers (Promega, Madison, U.S.A.) in a total volume of 28 µl at 70 °C for 5 min. After cooling on ice, reverse transcription was performed with 100 U M-MLV Reverse transcriptase (RNAse H Minus point mutant, Promega, Germany) for 50 min at 55 °C. cDNA samples were stored at − 20 °C. **qPCR quantification**. For quantification of total ATP7B transcript, a predesigned TaqMan qPCR probe spanning exons 4–5 was used (assay #Hs00167339_m1, Applied Biosystems, Freudenstadt, Germany). For testing various locations (− 70/− 130/− 300/− 550 bp) of the ATP7B promoter, we used the gene expression custom assay design tool with the appropriate sequence fragments of the ATP7B genomic reference sequence of the promoter region for generating TaqMan probes. qPCR context sequences are shown in Supplementary Table 1. All hydrolysis probes contained FAM as reporter dye and a non-fluorescent quencher. All used qPCR probes showed similar sensitivity as determined by a calibration qPCR with serial dilutions of the calibration plasmid (see above) over several orders of magnitude (Supplementary Fig. 1). However, the calibration plasmid containing both the ATP7B promoter as well as exon 4–5 was always quantified in parallel for normalizing all signals to a reference containing identical DNA copy numbers for each qPCR. Control qPCR reactions with DNAase I-digested RNA samples which had not been reverse transcribed were performed to validate that no amplification occurs. Non-template controls were always performed in parallel. qPCR reactions contained 10 µl total volume with TaqMan universal PCR master mix, the assay mixture containing primers and hydrolysis probe, and 5 µl sample. Cycling conditions were: 2 min 50 °C, 10 min 95 °C, 60 cycles with 15 s 95 °C and 60 s 60 °C. All tests were run in a StepOne qPCR machine (Applied Biosystems, Weiterstadt, Germany). The StepOne 2.0 software was used to measure qPCR curves. Exported Cq values were further analyzed in Excel. All Cq values that were used for calibration or for quantification were clearly below 40.

For assessing the relative abundance of transcript containing the different sequence motifs from the 5’-untranslated region of ATP7B, all Cq values were first normalized to the signal of the internal calibration plasmid (ΔCq(Calibration)). In a second step, they were related to the signal of the ATP7B exon 4–5 area (ΔΔCq(e45)). Factors were determined using the standard formula 2-ΔΔCq.

### Transfection

For transfection, cells were seeded at approximately 70% density into 96-well-plates (24.000 cells per well with 100 µl medium without antibiotics). Cells were transfected using Lipofectamine 2000 (Invitrogen, Waltham, U.S.A). Co-transfections were performed using pGL4.74 together with pGL4.10 with or without insertions of appropriate promoter sequences of ATP7B, each with 50 ng per well. Moreover, one co-transfection with an EGFP vector was performed in parallel for quick visual assessment of transfection efficiency. All plasmid preparations used were diluted with water to a final DNA concentration of 100 ng/µl to enhance precision of pipetted volumes. Master mixes were prepared of DNA and Lipofectamine, each diluted in Optimem according to the manufacturers’ recommendations. After incubation times as specified by the manufacturer’s instructions, both solutions were combined to achieve another master mix corresponding to a multiplicity of a final amount of 100 ng DNA and 0.2 µl Lipofectamine in 10 µl per well. Transfections were then performed with these mastermixes in triple or quadruple.

### Luciferase assay

48 h after transfection, luciferase expression was assessed using the Dual Glow Luciferase Assay System (Promega, Wisconsin, U.S.A.). Culture medium in the wells was replaced by 30 µl fresh medium. Firefly-luciferase reagent was added (25 µl), rocked and incubated for 10 min. Firefly luciferase activity was measured in a Perkin Elmer Envision analyzer equipped with luminescence filter (700 nm) and optical assembly. After detection of the firefly signal, 25 µl Stop-and-glo reagent was added, rocked and incubated for 10 min. Renilla signal was then measured. Reporter signal was calculated by dividing firefly signal by renilla signal, multiplicated with 100. In each experiment, 4–8 single, identically transfected wells were measured. Experiments were performed 2–10 times for each variant. Averages and standard deviations were calculated for each variant. Measured luciferase activities correlated well with the amount of used plasmid in test transfections (Supplementary Fig. 3).

### Bioinformatics analyses

For analyzing the promoter conservation of ATP7B, the genetic alignment of 91 mammals from the Ensembl platform was used. This alignment was applied to a ConSeq analysis^46^ which yields a score for each base of the human sequence from 1 (no conservation) to 9 (highly conserved). For clarity in a graphical presentation, the moving average of 10 periods was created for these conservation scores (Fig. 1B). Potential transcription initiation sites were retrieved from the literature^11^ and from three databases of transcriptional start sites: the Database for Transcriptional Start Sites (DBTSS)^25^, the Fantom5 database and the Eukaryotic Promotor Database (EPD) were used for annotating potential transcription initiation sites.

## Supplementary Information

Supplementary Figures and Tables
