# Supplementary material for: Investigation of the Wilson gene ATP7B transcriptional start site and the effect of core promoter alterations
Source: Sci Rep. 2021 Apr 7;11:7674. doi: 10.1038/s41598-021-87000-9 (PMC8027023; doi:10.1038/s41598-021-87000-9)
Supplement: Supplementary file 1 — Supplementary Figures and Tables [file 41598_2021_87000_MOESM1_ESM.pdf]

SUPPLEMENTARY INFORMATION

Investigation of the
Wilson gene ATP7B transcriptional start site
and the
effect of core promoter alterations

Clemens Höflich [1], Angela Brieger [1], Stefan Zeuzem [1], Guido Plotz [1]

[1] Biomedical Research Laboratory, 1st Department of Internal Medicine, University Hospital Frankfurt, Germany

Supplementary Table 1:

pGL4.10-ATP7B plasmid variants and primer sequences

pGL4.10-ATP7B vector/identifier		Genotype alterations in comparison to ATP7B reference sequence NG_008806:4024-5149 *	Site-directed primers used for generation
-54G>T	D1	rs115564351	5'-CGACCAGTTGACCTTTTGCTCTGAGCCAGATCAGAGAA<br>5'-AAAGGTCAACTGGTCGGTGGAGGAGAGCGGGGT
-36C>T	D2	rs762339422	5'-GCTCTGAGTCAGATCAGAGAAGAATTCGGTGTCCGT<br>5'-TGATCTGACTCAGAGCAAAAGGTCACCTGGTCGGTG
-32A>G	D3	rs759260854	5'-CTGAGCCAGGTCAGAGAAGAATTCGGTGTCCGT<br>5'-TGACCTGGCTCAGAGCAAAAGGTCACCTGGTCG
REFERENCE SEQUENCE	RR	None	Both primer pairs from RP and PR
Polymorphism -75 rs2277448	RP	rs2277448	5'-AGAGCCGAGCCGCGCCGATGCCCTCACACTCTGCGCCTC<br>5'-GAGGCGCAGAGTGTGAGGGCATCGGCGCGGCTCGGCTCT
Polymorphism -123 rs148013251	PR	rs148013251	5'-CTCCCGGGACTTTAACACCCCGCTCTCCTCCACCGAC<br>5'-GTCGGTGGAGGAGAGCGGGGTGTTAAAGTCCCGGGAG
Both polymorphisms	PP	rs148013251<br>rs2277448	originally cloned ATP7B sequence

* All preparations also contained rs1427836170, a single-nucleotide exchange far from the ATP7B translational start (-842 bp), but close to the ALG11 translational start codon. This alteration was tested not to influence luciferase reporter expression from the ATP7B promoter (data not shown).

Supplementary Table 2:

qPCR probe context sequences

qPCR assay	Context sequence (provided by manufacturer)
ATP7B-exon 4-5	CATTGAGCTGACAATCACAGGGATG
ATP7B-70	GGACTTTAACACCCCGCTCTCCTCC
ATP7B-130	TCCCGGACCCCTGTTTGCTTTAGAG
ATP7B-300	GCCACCTGGGGAGTGGGCGAGGGTC
ATP7B-550	CCGCAGGCGGTGGGTGAGCCCTGGG

Supplementary Table 3:

Genotype of the two ATP7B promoter polymorphisms in ancient and present genomes.

Sample	Identifier	Origin	Age	rs148013251	rs2277448	Coupled/ Uncoupled?*
Altai		*Neandertal*, Sibiria	90.000 years	Ref. seq.	Ref. seq.	n.a.
Chagyrskaya		*Neandertal*, Sibiria	60.000 years	Ref. seq.	Ref. seq.	n.a.
Vindija		*Neandertal*, Croatia	30.000 years	Ref. seq.	Ref. seq.	n.a.
Denisova		*Denisovan*, Sibiria	40.000 years	Ref. seq.	Ref. seq.	n.a.
Ust-Ishim	PRJEB6622	*Homo sapiens*, Sibiria	45.000 years	Ref. seq./rs148013251	rs2277448	n.a.
San	SS6004473	Africa	Present day	Ref. seq.	Ref. seq.	n.a.
Dinka	SS6004480	Africa	Present day	Ref. seq.	Ref. seq.	n.a.
Mandeka	SS6004470	Africa	Present day	Ref. seq.	Ref. seq.	n.a.
Mbuti	SS6004471	Africa	Present day	Ref. seq.	Ref. seq.	n.a.
Yoruba	SS6004475	Africa	Present day	Ref. seq./rs148013251	Ref. seq./rs227748	4/0
French	SS6004468	Europe	Present day	Ref. seq./rs148013251	rs2277448	n.a.
Sardinia	SS6004474	Europe	Present day	Ref. seq./rs148013251	Ref. seq./rs227748	6/0
Dai	SS6004467	East/Southern Asia	Present day	Ref. seq./rs148013251	rs2277448	n.a.
Han	SS6004469	Asia	Present day	Ref. seq./rs148013251	rs2277448	n.a.
Papua	SS6004472	Asia	Present day	Ref. seq.	Ref. seq.	n.a.
Australia	SS6004477	Oceania	Present day	Ref. seq./rs148013251	Ref. seq./rs227748	1/0
Australia	SS6004478	Oceania	Present day	Ref. seq./rs148013251	Ref. seq./rs227748	3/0
America	SS6004479	America Mixe	Present day	Ref. seq./rs148013251	Ref. seq./rs227748	4/0
Karitiana	SS6004476	South America indigenous	Present day	Ref. seq./rs148013251	Ref. seq./rs227748	4/0

* Sequences in which rs148013251 occurs together with rs2277448 (coupled)/sequences in which rs148013251 occurs with reference sequence in the position of rs2277448

Supplementary Table 4:

Results of the ElemeNT analysis of the core promoter

Retrieved in December 2020 from:
http://lifefaculty.biu.ac.il/gershon-tamar/index.php/element-description/elementv2

Input sequence:
ggtcccaaatgaaggggcggttcccggacccctgtttgctttagagccga
gccgcgccgatgccctcacactctgcgcctcctctcccgggactttaaca
ccccgctctcctccaccgaccaggtgaccttttgctctgagccagatcag
agaagaattcggtgtccgt

RESULTS:
No combinations of core promoter elements were detected in the input sequence.

Elements found:

Element Start	position	Sequence	PWM score	Consensus Match
BRE upstream	52	ccgcgcc	0.7334	7 out of 7
BRE upstream	47	ccgagcc	0.2043	6 out of 7
Mammalian Initiator	142	ccagatc	0.4262	7 out of 7
Mammalian Initiator	66	tcacact	0.2182	7 out of 7
Mammalian Initiator	5	ccaaatg	0.0739	6 out of 7
Mammalian Initiator	103	ccgctct	0.0667	6 out of 7
Mammalian Initiator	68	acactct	0.0600	6 out of 7
Mammalian Initiator	24	ccggacc	0.0474	6 out of 7
Mammalian Initiator	120	ccaggtg	0.0341	5 out of 7
Mammalian Initiator	96	taacacc	0.0260	6 out of 7
Mammalian Initiator	41	ttagagc	0.0244	6 out of 7
Mammalian Initiator	57	ccgatgc	0.0226	5 out of 7
Mammalian Initiator	52	ccgcgcc	0.0202	5 out of 7
Mammalian Initiator	98	acacccc	0.0182	5 out of 7
Mammalian Initiator	147	tcagaga	0.0170	5 out of 7
Mammalian Initiator	47	ccgagcc	0.0164	5 out of 7
Mammalian Initiator	132	ttgctct	0.0152	6 out of 7
Mammalian Initiator	36	ttgcttt	0.0152	6 out of 7
Mammalian Initiator	159	tcggtgt	0.0125	5 out of 7
Mammalian Initiator	17	gcggttc	0.0104	5 out of 7

There are many factors affecting transcription.
Please note that the putative motifs are only based on DNA sequence match.

Supplementary Figure 1: qPCR probe calibration

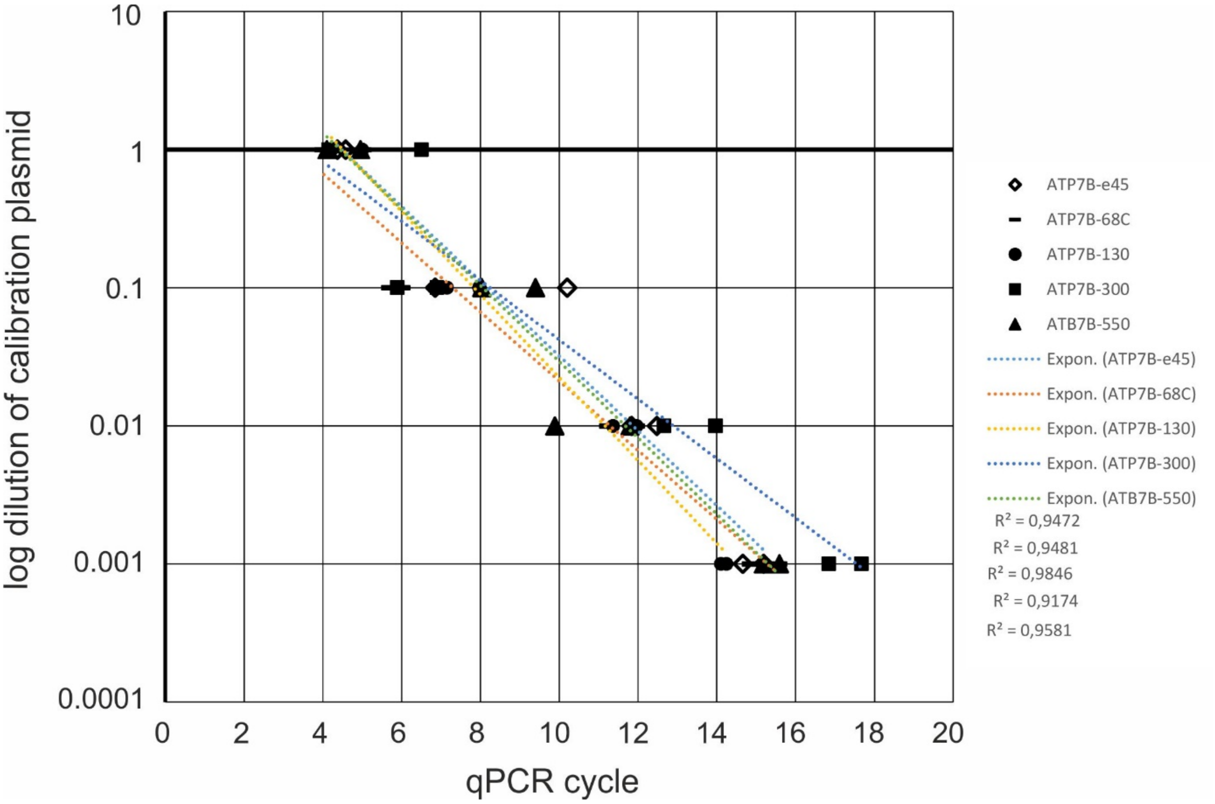

qPCR reactions were performed as detailed in Materials and Methods using dilutions of the calibration plasmid shown in **Figure 1C**. Ct values of the individual probes are shown. Linear regression lines were calculated using excel. Correlation coefficients are given.

Supplementary Figure 2: Frequency of the two promoter polymorphisms

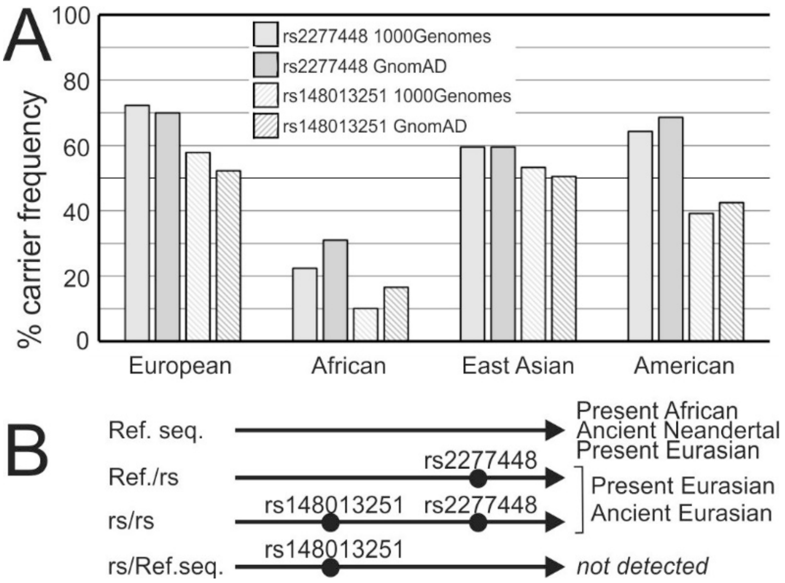

A. Data on carrier frequencies were retrieved in september 2020 from the assemblies of the NCBI dbSNP database.

B. The diagram demonstrates which combinations of rs148013251 and rs2277448 have been detected in ancient and present genomes of individuals of different geographical origin and is a graphical representation of the information in Supplementary Table 3. Ref.seq. or Ref. means reference sequence, rs means “reference SNP”.

Supplementary Figure 3: Linearity of luciferase assay measurements

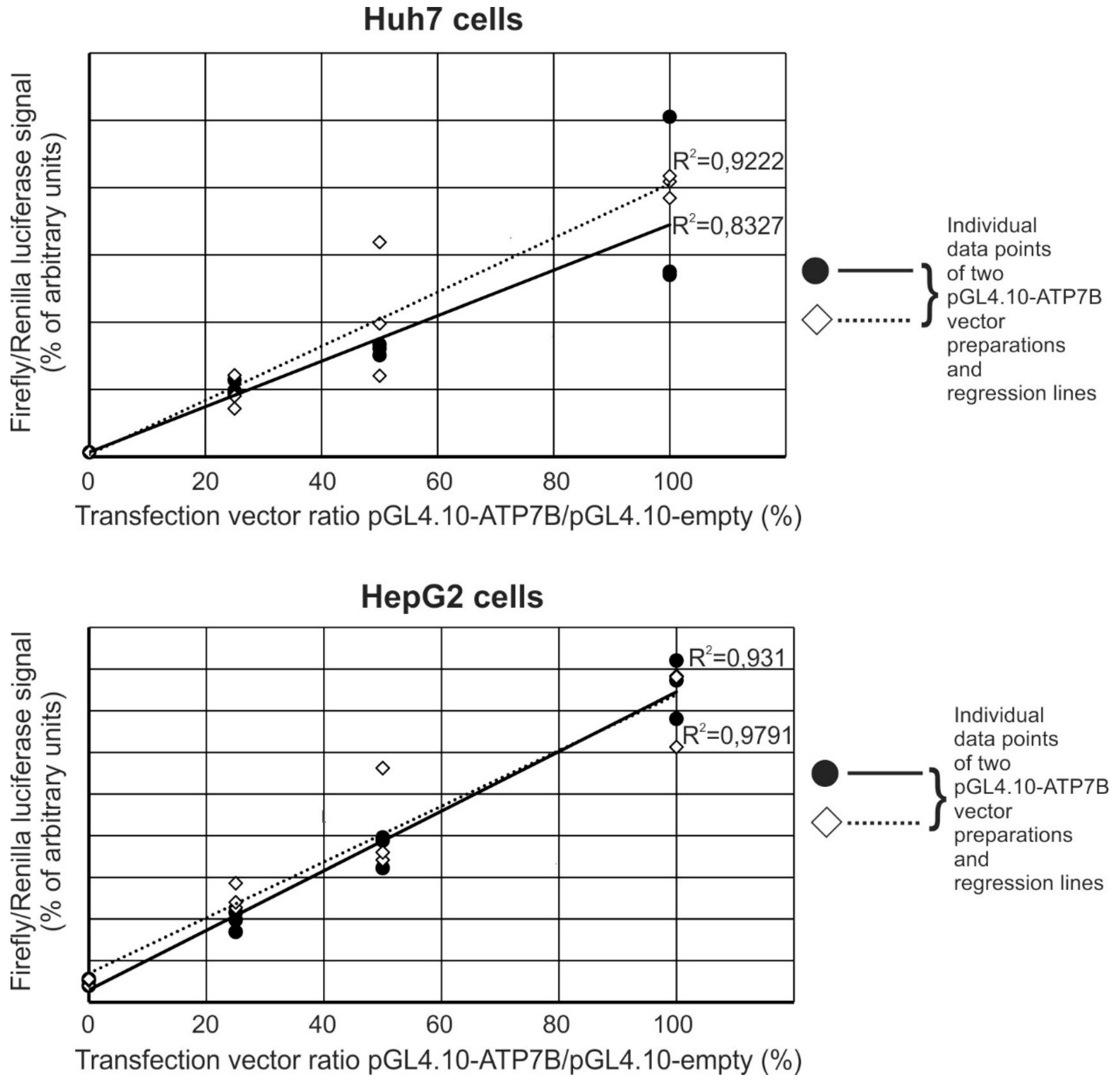

Huh7 or HepG2 seeded to 24.000 cells/well in 96-wells were co-transfected with 50 ng pGL4.74 (renilla control) and 50/25/12.5 ng pGL4.10-promoter vector (firefly probe) (100/50/25%), supplemented by 0/25/37.5 ng pGL4.10 vector (empty) to achieve 100 ng total plasmid DNA per well. All experiments were performed in triplicate. For control of transfection efficiency, a cotransfection of 50 ng pGL4.74 with 50 ng pcDNA3-EGFP performed in parallel. After 48 hours, transfection efficiency was about 40% as determined by fluorescence microscopy. Luciferase activities were measured as detailed in Materials and Methods. Regression lines and coefficient of determination R^2 are given.
